# Promoting intergroup inclusion through literary discussions in fourth and fifth grades

**DOI:** 10.1007/s11218-026-10179-0

**Published:** 2026-02-27

**Authors:** Luciano Gasser, Anna Frei, Yvonne Dammert, David Preisig, Sara Egger

**Affiliations:** Institute for Research, Development, and Evaluation, Bern University of Teacher Education, Bern, Switzerland

**Keywords:** Literary fiction, Intergroup attitudes, Classroom norms, Social inclusion, Migration background, Dialogue

## Abstract

The study investigated the effects of a one-year literary program on children’s intergroup attitudes toward peers with a migration background and on their perceptions of inclusive classroom norms. Fourth- and fifth-grade students and their teachers discussed four literary fiction books in small groups, with children taking the lead by asking their own questions, engaging in dialogue, and challenging one another. The books addressed topics such as intergroup exclusion, inclusive group norms, and civil courage. A total of 744 children participated in pre- and posttest assessments. The 41 classrooms were randomly assigned to either the intervention or the control group. Children’s intergroup attitudes were assessed using measures of intended intergroup contact, sympathy, and peer nominations indicating preferences for outgroup classmates. Inclusive classroom norms were assessed by having children rate their classmates’ intergroup-inclusive behaviors and fairness. Multilevel analyses revealed positive effects of the literary program on most intergroup attitudes and inclusive norms. However, these effects were small and emerged primarily among specific subgroups (i.e., children without a migration background, and in the younger grade level). We discuss the opportunities and challenges of using literary education as an integrated approach to promote both language development and social inclusion in schools.

**Supplementary Information:**

The online version contains supplementary material available at https://doi.org/10.1007/s11218-026-10179-0.

## Introduction

One of the greatest challenges for modern societies is to engage constructively with diversity—seeing differences as a source of creativity rather than as a threat to status and identity (Hundschell et al., 2022; Killen & Rutland, 2011). In Switzerland, however, discrimination—especially toward peers with a migration background—is increasingly reported in schools (Federal Commission against Racism, 2024; Federal Statistical Office, 2025). Such experiences undermine children’s mental health and academic development (Benner et al., 2018; Schmitt et al., 2014).

While most prevention programs to promote positive intergroup attitudes and norms are designed as extracurricular programs (Cameron et al., 2006; Mäkinen et al., 2019), few initiatives foster social inclusion within the regular curriculum. We test whether a yearlong literature-based intervention integrated into regular instruction improves intergroup attitudes toward children with a migration background and inclusive classroom norms among fourth and fifth graders.

### Intervention studies on children’s intergroup attitudes

Meta-analytic evidence indicates that children’s inclusive attitudes and behaviors can be improved through various strategies (Beelmann & Heinemann, 2014; Ülger et al., 2018). First, strategies that promote perspective-taking, sympathy, or moral reasoning are effective (Berger et al., 2016; Brenick et al., 2019; Gabrielli et al., 2022). Developmental research shows that when children adopt the perspective of outgroup members, they perceive more self-other similarity and individuate others rather than categorizing them by group membership (Aboud, 2008nültaş & Mulvey, 2021; Miklikowska, 2018; Pettigrew & Tropp, 2008). Moreover, moral reasoning helps children recognize and weigh the harmful consequences of discrimination (Gasser et al., 2017; Killen & Rutland, 2011).

Second, strategies that facilitate positive intergroup contact enhance children’s social inclusion, for example by supporting cross-group learning or friendships (Gasser et al., 2014; Grütter et al., 2018; Van Ryzin et al., 2020). More recently, studies have examined vicarious contact whereby prejudice is reduced when positive intergroup contact is experienced indirectly through games, films, or stories (Paluck et al., 2021; Stathi, 2017; Vezzali et al., 2012; White et al., 2021). The effects are attributed to social learning processes through which children, by immersing themselves in fictional intergroup narratives and witnessing inclusive behaviors, adopt more positive attitudes toward outgroup members.

In vicarious-contact interventions, researchers often develop tailored stories and discussion guidelines to ensure that children engage in focused reflections that destabilize intergroup bias (Cameron et al., 2006, 2011; Cocco et al., 2021; Mäkinen et al., 2019, 2024). For example, Killen et al. (2022) created an interactive web-based curriculum (“Developing Inclusive Youth”) featuring eight short stories about intergroup exclusion. Each story presents one protagonist who endorses exclusion using stereotypes and another who rejects exclusion for moral reasons. After listening and interacting with the stories, teachers facilitate discussions that prompt children to reflect on the harmful consequences of intergroup exclusion, connect the stories to their own experiences, and consider both group-based and moral concerns. A randomized controlled trial demonstrated higher moral reasoning and fewer stereotypes in the intervention group compared to controls. Thus, reading and discussing stories about intergroup conflicts is a promising strategy for promoting children’s social inclusion.

### Intervention programs that integrate social and academic learning

A key advantage of vicarious-contact interventions is their high level of standardization through researcher-designed stories and discussion guidelines. However, such standardization can also hinder implementation in schools (Mäkinen et al., 2022). First, most curricula define aesthetic education as a core learning objective (Witte & Sâmihaian, 2013). Reading literary fiction substantially contributes to this goal (Kumschick et al., 2014; Schrijvers et al., 2019). In contrast, researcher-developed intergroup stories may be less appealing to children and teachers and thus yield smaller social benefits (Vezzali et al., 2015). Literary fiction more readily elicits narrative transportation—a deep imaginative immersion into the story world—which facilitates perspective-taking and attitudinal change by allowing readers to experience stories through the protagonist’s eyes (Johnson, 2012; Koopman, 2018; Oľhová et al., 2023). Second, teachers often use stories to promote language learning (e.g., vocabulary or text comprehension; Sverdlov et al., 2014). When intergroup stories are framed only as tools for promoting social inclusion and decoupled from academic learning goals, teachers’ motivation to adopt these approaches declines (Elias et al., 2000; Jones et al., 2011; Nucci & Ilten-Gee, 2021).

The national curriculum in Switzerland (D-EDK, 2016), as well as those in many other countries in Europe (European Education and Culture Executive Agency, 2023), explicitly expects teachers to promote children’s social skills—including the ability to effectively engage with diversity—by creating social learning opportunities within subject-specific instruction. However, few prevention programs provide actionable guidance for facilitating such social learning in regular lessons (Kochenderfer-Ladd & Ladd, 2016). The 4Rs program uses high-quality children’s books to link social and academic learning and has shown gains in children’s social cognition and social behavior (Doyle et al., 2023; Jones et al., 2010, 2011). The Collaborative Social Reasoning (CSR) program fosters peer discussions about stories that combine cognitive depth with inclusive, equitable participation, with positive effects on argumentative writing, perspective-taking, and social behavior (Lin et al., 2019, 2022; Wen et al., 2023).

Despite promising evidence from integrated programs, the potential of literary education to foster inclusive attitudes and classroom norms within regular instruction remains underexamined. Only a few short-term experiments have explored this, typically using brief excerpts from literary works (e.g., *Harry Potter* passages) outside of a broader instructional context (Oľhová et al., 2023; Vezzali et al., 2015). Although effective in controlled settings, such formats have limited ecological validity and curricular integration, which hinder their implementation in everyday classroom practice.

### The literary intervention of this study

Based on Louise Rosenblatt’s (1978) transactional reading theory, a recent literature review identified three approaches to how children learn socially from literary fiction (Gasser et al., 2022). The *efferent approach* assumes social learning via moral messages that readers retain after reading. The *expressive approach* focuses on the spontaneous and affective reactions while reading; when immersed in fictional worlds, readers can train their perspective-taking and sympathy. The *critical-analytic approach* locates social learning in the epistemic triangle of child, co-readers, and text. Through dialogic discussions with peers, teachers, or parents, children justify their interpretations, listen to others, and integrate others’ perspectives—moving beyond a simple internalization of moral messages and affective responses toward well-examined interpretations of the story.

Our intervention adopts the Quality Talk (QT) model, which exemplifies a critical-analytic approach to social learning from literary fiction (Gasser et al., 2022; Murphy et al., 2022). QT promotes critical thinking and high-level text comprehension through small-group discussions about texts. In these discussions, the teacher steps back, transferring interpretative authority and responsibility for discussion management (e.g., turn-taking) to the children. Instead, the teacher supports the discussions with discourse moves that enhance the quality of questions and answers (e.g., modeling open-ended questions, prompting for reasons, encouraging build-ons, inviting alternative perspectives). To prepare children for dialogic discussions, brief explicit instruction introduces authentic question types (e.g., affective, or intertextual questions) and elements of effective responses (e.g., how to respectfully disagree). Across the school year, children read and discuss four children’s books addressing social exclusion, intergroup conflict, and civil courage. Each book features a positive protagonist who models inclusive behavior toward outgroup members. Before each book, children were introduced to the social topic of the book (e.g., stereotypes) to provide the necessary conceptual knowledge and vocabulary for reading and discussion.

### The present study

**Fig. 1:**
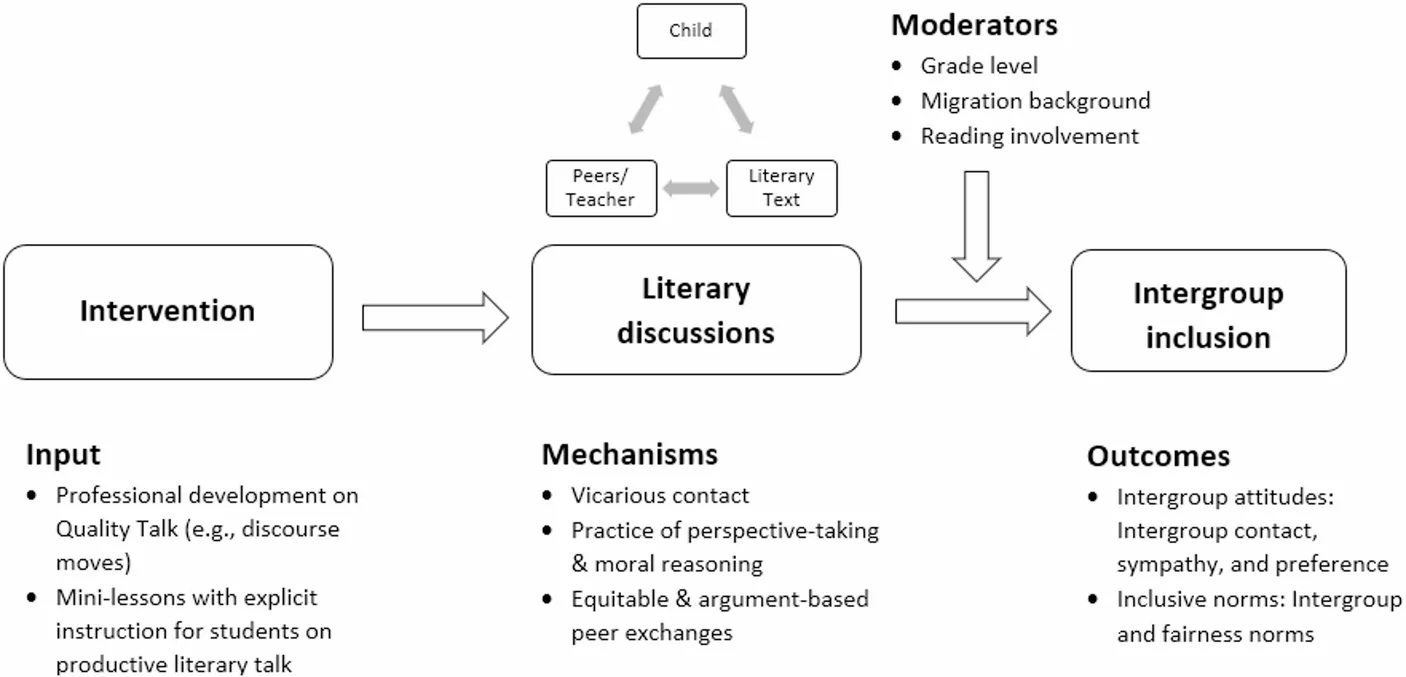
Theory of change of intergroup inclusion

We expect our intervention to influence children’s social inclusion through three mechanisms (Fig. 1): (1) *Vicarious contact*. Stories with high literary quality (e.g., outstanding language, creativity, complex characters) encourage immersive experiences, enabling children to vividly imagine how story protagonists combat prejudice, celebrate diversity, and demonstrate civil courage; (2) *Practice of perspective-taking and moral reasoning*. Through literary discussions with peers, children gain extended opportunities to put themselves into the shoes of story characters and discussion partners (Oľhová et al., 2023). Moreover, the stories present complex social cases of inclusion and exclusion, allowing practice of contextualized moral judgments (Killen et al., 2022); (3) *Promotion of inclusive group norms.* Collaborative peer discussions, characterized by argumentation and equity, establish inclusive norms that ensure that all perspectives in the discussion are heard and valued (Chen et al., 2023; Gasser & Althof, 2017; Habermas, 2016). Such norms discourage social hierarchies and discrimination in peer groups, while fostering cooperation and social inclusion (Gasser et al., 2017, 2018; McGuire et al., 2019).

The study focuses on children in the fourth and fifth grades because developmental research has shown that this age is a critical phase in the development of outgroup attitudes (Killen & Rutland, 2011). With growing sensitivity to peer group norms, older children increasingly face a conflict between loyalty to their peer groups and awareness of the injustice of intergroup exclusion. This heightens support of bias-based exclusion and bullying in late childhood and early adolescence (Mulvey, 2016). Consequently, it is important to strengthen the inclusive capacities of children in late childhood and early adolescence.

This study uses two types of child outcomes: (1) *Intergroup attitudes* (intended intergroup contact, intergroup sympathy, outgroup peer preference), and (2) *Inclusive norms* (intergroup norm and fairness norm). While self-rated intergroup attitudes are commonly used outcomes in studies on stereotype reduction, peer nominations and perceptions of peer group norms are rarely employed (Paluck et al., 2021). As self-presentational concerns increasingly influence the expression of outgroup attitudes in late childhood (Monteiro et al., 2009; Rutland et al., 2005), it is important to include measures less susceptible to social desirability. These include, for example, how much children like or dislike outgroup classmates (i.e., outgroup peer preference), or how fairly they expect their peers to behave—both in general and specifically toward outgroup members (i.e., fairness norm and intergroup norm).

The attitudinal and group norm measures focus on inclusion of individuals with a migration background because discrimination against this target group is most frequent in Switzerland (Federal Statistical Office, 2025). Ratings of peers’ fairness tap more general inclusive capacities such as fair and respectful behavior because fairness represents an important resource in combating stereotypes and discrimination (Killen & Rutland, 2011).

We expect positive effects of the literary intervention on both intergroup attitudes and inclusive norms. Furthermore, grade level was considered a moderator of intervention effects, as previous research has found stronger effects of interventions targeting social inclusion or exclusion among younger than older children (Killen et al., 2022; Yeager et al., 2015). We also tested whether effects are stronger for children without a migration background than for those with a migration background because interventions more effectively improve inclusive attitudes among majority- than minority-group children (Ülger et al., 2018). Accordingly, moderation by migration background was also specified a priori as theory-driven. Finally, we included reading involvement as a covariate and examined its potential moderating role in an explicitly exploratory manner.

## Method

### Sample and study design

The sample consisted of 744 children (48.7% girls) from 18 fourth-grade classrooms (*M* = 9.83 years, *SD* = 0.50) and 23 fifth-grade classrooms (*M* = 10.74 years, *SD* = 0.51). A total of 30.8% of the children had two parents who were born outside of Switzerland, while 29.8% spoke a language other than German at home. Teachers, parents, and children provided written consent to participate in the study (rejection rate = 2.8%). The project was approved by the Ethics Committee of Bern University of Teacher Education.

The study followed a cluster-randomized controlled design with two measurement points: Pretest (*M* = September 2, 2022, *SD* = 9 days) and Posttest (*M* = June 13, 2023, *SD* = 11 days). Control classes followed business-as-usual language arts instruction and received no study materials or training. Classes were randomly assigned to either the control or intervention group before the start of the intervention, considering two stratification criteria: grade level (4th or 5th) and percentage of non-Swiss residents in the school’s communities. These criteria ensured that the grade levels and percentage of non-Swiss children at the school level were equally distributed across the intervention and control groups. One class from the control group dropped out of the study during the pretest, resulting in 21 classes in the intervention group and 20 in the control group. We did not have other dropouts at the class level. The intervention and control groups did not differ significantly in terms of their composition regarding children with Swiss German as their home language, χ^2^(1) = 0.00, *p* =.980, parents born outside Switzerland, χ^2^(1) = 0.00, *p* =.950, or grade level, χ^2^(1) = 1.45, *p* =.228. In 31 classrooms, only one teacher participated, while in 10 classrooms, teachers participated in pairs. Overall, 51 teachers took part in the study.

### Power analysis

Because prior work did not report effects of literary interventions on intergroup attitudes, the power analysis focused on students’ academic outcomes (e.g., reasoning). Effect sizes were taken from the meta-analysis of Murphy et al. (2009) and from primary studies, covering β = 0.03–0.87. We simulated two-level models in R (students nested within classrooms; α = 0.05), evaluating designs with 30 and 40 classrooms and 20 students per classroom. Across this effect-size range, a 40-classroom design yielded power of 0.79–0.95; the 30-classroom scenario did not reach conventional power (≈ 0.80). We therefore targeted 40 classrooms to detect small-to-large effects on student outcomes.

### Procedure

The two assessments were conducted during a school morning by two trained research assistants (10 h of training). The participants received standardized instructions from the research assistants (e.g., regarding the procedure and material handling). Privacy screens were used to ensure that the children could complete the tasks and questionnaires independently. The participants filled out a battery of questionnaires and tasks on tablets using the survey software SurveyCTO.

### Measures

#### Covariates

*Migration background* Children indicated whether each of their parents was born in Switzerland. In reference to the typology of the Swiss Federal Statistical Office (SFSO, 2024), we defined migration background as having both parents born outside of Switzerland. This conservative operationalization captures a subgroup with a clear and consistent family-level migration context. The child’s own place of birth or citizenship was not considered, as the focus was on familial migration-related socialization. According to this definition, 30.8% of the children were classified as having a migration background.

*Reading involvement* We used a subscale from the *Reading Motivation Questionnaire for Elementary Students* (RMQ-E; Schiefele & Löweke, 2018) to assess children’s involvement in reading (i.e., “I read because books and stories are often fascinating,” “I read because it’s fun,” “I read because it’s exciting to see what happens to the characters in a story”). Children rated the items on a 4-point scale (1 = *does not apply at all* to 4 = *fully applies*). The scale was administered only at posttest. Internal consistency was acceptable (Cronbach’s α = 0.80), and a mean score was computed across the three items.

#### Intergroup attitudes

*Intended intergroup contact* To assess intended intergroup contact, we first asked the participants to imagine that a child from another country joined their class who did not speak German very well yet (Gasser et al., 2018). Children were next asked how much they would like to engage in five different activities with the hypothetical child (e.g., “How much would you like to play with this child?”, “How much would you like to celebrate a birthday party with this child?”). Children rated their intentions for intergroup contact on a 4-point scale (1 = *not at all* to 4 = *very much*). Cronbach’s *α* was 0.86 for pretest and 0.87 for posttest scores. The five items were averaged to build a mean score.

*Intergroup sympathy* Following the items on intended intergroup contact, children were asked to imagine how sorry they would feel for a hypothetical child experiencing various forms of social exclusion (e.g., “How sorry would you feel for this child if the child is being bullied?”, “How sorry would you feel for this child if the child doesn’t have any friends?”). The three items were adapted from Grütter et al. (2018). The participants rated their sympathy on a 4-point scale (1 = *not at all* to 4 = *a lot*). Cronbach’s *α* was 0.90 for both measurement points. A mean score was calculated.

*Outgroup peer preference* Children were asked to nominate an unlimited number of classmates they liked (acceptance) and disliked (rejection). In this measure, we focused not on how many nominations a child received, but on how many nominations the child gave and to whom. When counting the number of positive and negative nominations, we only included nominations where the nominator and nominee differed in whether they had a migration background or not. More specifically, if the nominator had a migration background, only nominations of peers without a migration background were counted, and vice versa. Since children in classrooms with more outgroup classmates had a higher chance to nominate across groups, we standardized the number of positive and negative intergroup nominations by dividing each by the number of potential outgroup classmates. Potential nominees had to have no missing values on their migration background. To assess outgroup peer preference, we calculated a preference score by subtracting the standardized intergroup rejection score from the standardized intergroup acceptance score. Thus, higher scores indicate a more positive evaluation of outgroup classmates, whereas lower scores indicate a more negative evaluation. The outgroup peer preference score was computed separately for pre- and posttest.

#### Inclusive norms

*Intergroup norms* Children’s perceptions of intergroup norms were assessed using a scale developed by Gasser et al. (2018). After children rated their intended contact and sympathy toward a hypothetical child with a migration background, they were asked to indicate how many of their classmates would help if the child were socially excluded (e.g., “How many children in your class would help this child if the child were being bullied by older kids?,” “How many children in your class would help this child if the child were often lonely during recess?”). Children rated how many of their classmates would help the child on a 4-point scale (1 = *none* to 4 = *all*) across four items. Cronbach’s α was 0.77 for the pretest and 0.82 for the posttest. Item scores were averaged to create a total mean score.

*Fairness norm* Children rated their classmates’ fairness based on four items that described respectful, caring, cooperative, and helping behaviors (e.g., “Students in this classroom really care about each other,” “Students in this classroom treat each other with respect”). The items were adapted from Ding et al. (2011). Items were rated on a 4-point Likert scale (1 = *strongly disagree* to 4 = *strongly agree*). The scale showed good internal consistency (Cronbach’s α at pretest = 0.83 and at posttest = 0.85). A mean score was calculated.

### Implementation of the intervention

**Fig. 2:**
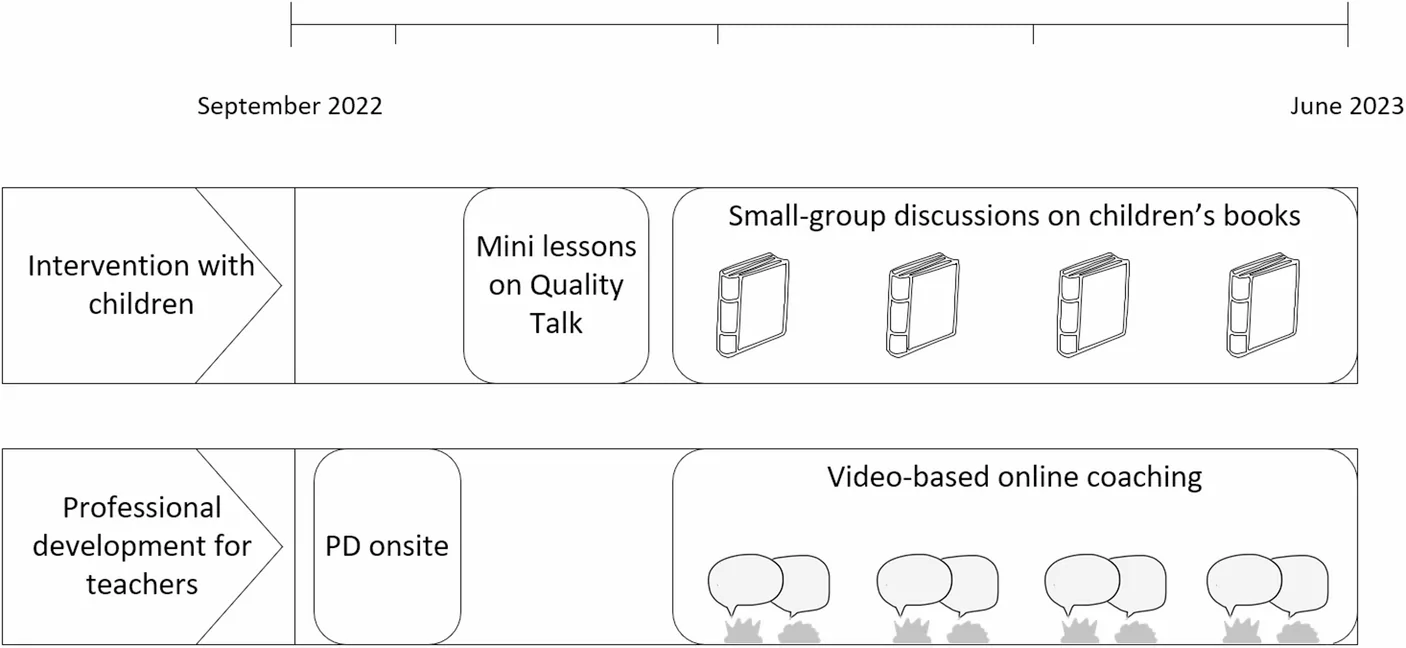
Intervention design

An overview of the intervention is displayed in Fig. 2. The professional training for teachers included two elements (for more details on the intervention, see Sect. 1 in the online Appendix). First, teachers participated in a *two-day training* session led by the study team after the pretest assessment (September 2022). This training had a dual focus: (1) instruction on facilitating literary discussions based on the Quality Talk approach, and (2) an introduction to the social concepts embedded in the children’s books, including insights into how children in this age group perceive these issues. After the training, teachers implemented the intervention in their classrooms and received three to four *video-based online coaching* sessions from the research team to support high-quality literary discussions (number of coaching sessions: *M* = 3.11, *SD* = 0.79). Teachers shared videos of their discussions with coaches, who assisted them in enhancing their observation and reflection skills on the socioemotional and instructional quality of interactions during literary discussions.

The intervention in classrooms comprised two types of lessons. In the first two weeks, teachers conducted nine *mini lessons* aimed at introducing children to effective questioning and responding techniques. For example, children learned to use various types of open-ended, authentic questions (e.g., affective questions that prompt personal connections with the story), empowering them to ask their own questions during discussions. Using a PowerPoint presentation, teachers first defined and explained a specific discourse element. They then showed and discussed an animated video modeling the use of the discourse element with the children, followed by practice (e.g., identifying an authentic question from a comic movie).

Following the mini lessons, *book lessons* began, in which children read and discussed four fiction books (see Sect. 2 in the online Appendix for a summary of the books). Before starting each new book, children participated in instructional activities designed to introduce relevant social vocabulary and conceptual knowledge on the book’s theme (e.g., the role of group norms in social inclusion, nature of prejudice, civil courage). Discussions occurred in small groups facilitated by the teacher. Over the school year, children participated in an average of 9.10 small-group discussions.

Teachers received various materials to support the standardized implementation of the intervention in their classrooms (e.g., detailed lesson plans; for sample materials and small-group discussions, see https://www.phbern.ch/skill). Teachers reported that they invested 33.03 h for the intervention (*SD* = 6.27, range = 12.58 to 42.00 h), with 23% allocated to mini lessons and 77% to the book lessons. Teachers rated their adherence to lesson plans on a 4-point scale (0 = *never* to 3 = *frequently*) and indicated minimal deviation from the plans (*M* = 1.05, *SD* = 0.19). Thirteen classes read and discussed three children’s books, while seven classes engaged with all four books. One teacher implemented less than 30% of the intervention (covering only the mini-lessons and one book, without participating in coaching). However, following the Intention-to-Treat (ITT) principle, this classroom was retained in the analyses. The ITT approach entails including all originally assigned units (in this case, classrooms) in the final analysis, regardless of deviations from the assigned condition or implementation issues. This method preserves the initial randomization and prevents potential bias arising from post-randomization exclusions (Gupta, 2011).

### Statistical analyses

Due to the hierarchical structure of our dataset, where children are nested within classrooms, we first computed unconditional models for the five outcome variables to examine the intraclass correlation coefficients (ICC). The ICC was 10.8% for intended intergroup contact, 2.3 % for intergroup sympathy, 5.3 % for peer preference, 13.1% for intergroup norm, and 17.0% for fairness norm.

Given this clustering, we employed two-level analyses of covariance (ANCOVA) to predict posttest outcomes while controlling for pretest scores. Although the ICC for intergroup sympathy was low, we decided to apply two-level models for all outcomes to maintain methodological consistency. Given the low variation in class size (*M* = 19.63, *SD* = 2.83, range: 13–25; coefficient of variation: 14.4%) and the minimal, random nature of missing data (see Sect. 2.6, “Missing Data”), two-level ANCOVA was preferred over change score models or unconstrained mixed models, as it offers greater statistical power under such conditions (Van Breukelen, 2023).

The analyses were performed with SPSS Version 29.2.0. At the child level, we included pretest scores, gender, migration background, and reading involvement as covariates. At the teacher level, grade and condition (intervention vs. control group) were included. We also tested whether the intervention effects were moderated by grade level (fourth vs. fifth grade), migration background, and level of reading involvement. These interaction terms were retained in the final model only if they reached statistical significance and were subsequently probed using simple slope analyses (Preacher et al., 2006). All continuous predictors (e.g., reading involvement, pretest scores) were grand-mean centered.

To evaluate model fit, we reported conditional R-squared values, reflecting the variance explained by both fixed and random effects, and marginal R-squared values, which indicate the variance explained by fixed effects only. Standardized effect sizes (Cohen’s *d*) were calculated by dividing the model-derived regression coefficients (*B*) by the standard deviation of the outcome variable—using subgroup-specific standard deviations for interaction effects and the overall standard deviation for main effects.

### Missing data

Multilevel ANCOVA models require outcome data at both measurement points. For all outcomes except outgroup peer preference, complete data were available for 669 children, meaning they had valid scores on all relevant predictors and outcome variables at both time points (1,338 data points in total). Across all participants, there were 725 valid pretest and 695 valid posttest scores, leading to a total of 1,420 potential data points. Based on this, the overall missing data rate was 5.77%. For the outcome outgroup peer preference, valid data were available for 637 children at both time points (1,274 out of 1,320 possible data points), resulting in a missing data rate of 3.48%. This outcome’s smaller sample size reflects the exclusion of an entire classroom with 21 children that contained no children with a migration background.

Systematic dropout was examined by comparing children with complete versus partial data, based on outcome scores from the available wave. Independent samples t-tests revealed no significant differences on any of the continuous pretest or posttest variables (all *p*s > 0.05). Furthermore, dichotomous predictors such as condition and migration background were not associated with data completeness. These findings suggest that the data were largely missing at random (MAR) and that attrition is unlikely to have biased the results.

## Results

### Descriptive statistics

Means, standard deviations, and correlations among Level 1 variables are presented in Table 1. Grade and Condition, both Level 2 predictors, were not significantly correlated. Importantly, significant correlations were found not only among self-reported intergroup measures, but also between peer-reported and self-reported measures (e.g., between outgroup peer preferences and intended intergroup contact).

### Main analyses

Table 2 presents the final models. Intercepts indicate expected posttest outcomes for fourth-grade girls in the control group, without a migration background, at average levels of reading involvement and average pretest scores.

#### Intergroup attitudes

*Intended intergroup contact* Children in the intervention group reported significantly higher levels of intended intergroup contact than those in the control group. The intervention effect was small (Cohen’s *d* = 0.19). Moreover, boys reported lower levels of intended contact than girls, and children with higher reading involvement or a migration background reported greater intentions for intergroup contact.

*Intergroup sympathy* A significant Condition × Migration Background interaction emerged. Simple slope analyses revealed a significant positive intervention effect for children without a migration background (*B* = 0.15, *SE* = 0.07, *p* =.029), but not for those with a migration background (*B* = −0.12, *SE* = 0.09, *p* =.183). The intervention effect for children without a migration background was small (Cohen’s *d* = 0.22). Additionally, reading involvement was positively associated with intergroup sympathy.

**Table 1:** Descriptive statistics for level 1 variables (means, standard deviations, correlations)

	*M (SD)*	1	2	3	4	5	6	7	8	9	10	11	12	13
1. Gender	0.52 (0.50)	-												
2. Reading involvement	3.08 (0.80)	− 0.21***	-											
3. Migration background	0.31 (0.46)	− 0.01	− 0.11**	-										
4. Intended intergroup contact T0	2.92 (0.67)	− 0.16***	0.06	0.09*	-									
5. Intergroup sympathy T0	3.34 (0.79)	− 0.15***	0.16***	− 0.08	0.20***	-								
6. Outgroup peer preference T0	− 0.02 (0.30)	0.01	0.08*	0.12**	0.11**	0.08*	-							
7. Intergroup norm T0	2.56 (0.58)	0.00	0.08*	0.07	0.27***	0.26***	0.19***	-						
8. Fairness norm T0	3.17 (0.60)	− 0.01	0.17***	− 0.08*	0.29***	0.13***	0.10*	0.37***	-					
9. Intended intergroup contact T1	2.76 (0.67)	− 0.22***	0.17***	0.13***	0.51***	0.16***	0.18***	0.20***	0.14***	-				
10. Intergroup sympathy T1	3.33 (0.70)	− 0.14***	0.21***	− 0.04	0.17***	0.40***	0.13***	0.19***	0.15***	0.31***	-			
11. Outgroup peer preference T1	−0.05 (0.34)	− 0.01	0.11**	0.10*	0.11*	0.08*	0.35***	0.09*	0.15***	0.17***	0.09*	-		
12. Intergroup norm T1	2.50 (0.61)	0.02	0.09*	0.02	0.20***	0.10**	0.15***	0.40***	0.26***	0.32***	0.35***	0.14***	-	
13. Fairness norm T1	3.00 (0.64)	0.01	0.20***	− 0.02	0.20***	0.05	0.19***	0.28***	0.47***	0.27***	0.17***	0.20***	0.43***	-

Note. T0 = pretest, T1 = posttest

^*^p <.05. ^**^p <.01. ^***^p <.001

**Table 2:** Estimates (and standard errors) of multilevel models predicting intergroup attitudes and inclusive norms

	Intergroup attitudes		Inclusive norms			
	Intended intergroup contact	Intergroup sympathy	Outgroup peer preference		Intergroup norm	Fairness norm
Fixed effects						
Intercept	2.75*** (0.06)	3.32*** (0.06)	−0.04 (0.03)		2.34*** (0.07)	2.97*** (0.07)
Pretest score	0.45*** (0.03)	0.32*** (0.03)	0.37*** (0.04)		0.37*** (0.04)	0.44*** (0.04)
Gender^a^	−0.16*** (0.04)	−0.09^†^ (0.05)	−0.00 (0.03)		0.04 (0.04)	0.04 (0.04)
Migration background^b^	0.15** (0.05)	0.13^†^ (0.08)	0.05^†^ (0.03)		−0.01 (0.05)	0.06 (0.05)
Reading involvement	0.11*** (0.03)	0.12*** (0.03)	0.00 (0.02)		0.04 (0.03)	0.09** (0.03)
Grade^c^	−0.04 (0.06)	−0.02 (0.06)	−0.04 (0.03)		0.09 (0.08)	−0.15* (0.07)
Conditon^d^	0.13* (0.06)	0.15* (0.07)	0.00 (0.03)		0.38*** (0.09)	0.14* (0.07)
Condition × Grade					−0.31** (0.11)	
Condition × Migration background		−0.26* (0.11)				
Condition × Reading involvement			0.06* (0.03)			
Random effects						
Residuals	0.30 (0.02)	0.39 (0.02)	0.10 (0.01)		0.28 (0.02)	0.28 (0.02)
Intercept (classroom)	0.02 (0.01)	0.01 (0.01)	0.00 (0.00)		0.02 (0.01)	0.03 (0.01)
Model summary						
BIC	1150.72	1316.07	382.94		1114.98	1119.93
Marginal/Conditional *R*^2^	0.30/0.34	0.19/0.21	0.14/0.16		0.20/0.24	0.24/0.31

Note. ^a^ 0 = girls, 1 = boys. ^b^ 0 = no migration background, 1 = migration background. ^c^ 0 = fourth grade, 1 = fifth grade. ^d^ 0 = control group, 1 = intervention group

^†^*p* <.10. *p <.05. ^**^p <.01. ^***^p <.001

*Outgroup peer preference* A significant Condition × Reading Involvement interaction was found. However, simple effects of condition at lower versus higher levels of reading involvement were not statistically significant. Probing the interaction by condition indicated that reading involvement was not associated with outgroup peer preference in the control group (*B* = 0.00, *SE* = 0.02, *p* = .892), but was positively associated in the intervention group (*B* = 0.07, *SE* = 0.02, *p* = .002). No additional significant effects were observed.

#### Inclusive norms

*Intergroup norm* A significant main effect of Condition was qualified by a Condition × Grade interaction. Simple slope analyses revealed a significant positive effect of the intervention for fourth graders (*B* = 0.38, *SE* = 0.09, *p* <.001), but not for fifth graders (*B* = 0.08, *SE* = 0.08, *p* =.335). The intervention effect size for fourth graders was moderate (Cohen’s *d* = 0.58).

*Fairness norm* A significant main effect of Condition indicated that children in the intervention group perceived their peers as fairer and more inclusive than those in the control group. The effect size was small (Cohen’s *d* = 0.22). Furthermore, children with higher levels of reading involvement reported more positive peer perceptions. Fifth graders, in contrast, perceived their peers as less fair than fourth graders.

## Discussion

In recent years, scholars have increasingly emphasized the importance of ecologically valid interventions to promote positive intergroup attitudes in children, adolescents, and adults (Paluck et al., 2019). A growing concern in this area is that many vicarious contact interventions are conducted in highly controlled, decontextualized settings, which limits their applicability in everyday educational contexts. Particularly, Mäkinen et al. (2022) have criticized the widespread practice of researcher-led interventions, arguing that such approaches often overlook the critical role of teachers as role models for inclusive behaviors in the classroom. Consequently, there is a pressing need to explore how teachers themselves can effectively implement strategies that foster positive intergroup attitudes and inclusive norms within the realities of their daily teaching practice.

Accordingly, a major goal of the current intervention study was to test the efficacy of teaching strategies that aim to integrate social learning about intergroup relationships into the academic curriculum. So far, most interventions on intergroup attitudes have been designed as extracurricular programs with no explicit connection to regular curricular activities. Even though some programs highlight the important role of teachers in creating inclusive classrooms, they seldom understand this task as an integral part of good and effective teaching.

An integrated approach to social and academic learning is not only responsive to teachers’ limited resources but also acknowledges the inherent interdependence between social, emotional, and cognitive learning processes in the classroom. For example, learning processes that support the development of social perspective-taking contribute not only to more inclusive attitudes and fairness (Gönültaş & Mulvey, 2021; Sobel & Blankenship, 2021), but also to better reading comprehension (Dore et al., 2018). Similarly, critical-analytic thinking helps prevent the development of prejudice (Hjerm et al., 2018) and supports high-level text comprehension (Murphy et al., 2009). By promoting perspective-taking and critical thinking in an academically relevant setting such as literary discussions, we naturally seized social learning opportunities in language arts to foster children’s intergroup attitudes and inclusive norms.

The study revealed positive intervention effects across most outcomes, but no overall between-group difference emerged for outgroup peer preference. Moreover, the effect sizes were small or emerged only in specific subsamples (i.e., fourth graders, children without a migration background). This contrasts with previous research showing stronger intervention effects of reading and discussing intergroup stories on children’s attitudes toward peers with a migration background (Cameron et al., 2006; Vezzali et al., 2012). One possible explanation for these weaker findings is that our intervention focused less explicitly on stereotypes toward a specific outgroup than other stereotype-reduction programs (e.g., Mäkinen et al., 2024). For instance, in Cameron et al.’s (2006) study, children discussed researcher-designed stories that all centered on intergroup friendships between children with and without a migration background. Researchers further often supported discussions based on predefined script that included specific prompts to enhance children’s awareness of characters’ group membership.

In our study, by contrast, children generated their own questions about the books and assumed interpretative authority over the texts, while teachers intervened as little as possible. We assumed that children’s intergroup attitudes and inclusive norms would change through improved rational discourse, perspective-taking, and critical thinking. Moreover, the selected books often addressed intergroup inclusion more generally and symbolically, without referencing specific outgroups. Even though Vezzali et al. (2015) showed that fictional stories such as Harry Potter can reduce negative attitudes toward immigrants, it remains possible that children’s books and discussions should more explicitly focus on specific outgroup targets to effectively enhance inclusive attitudes towards these groups. A recent intervention study found that intergroup bias decreased only when parents engaged their children in race-conscious conversations after watching an animated video depicting racial bias (Perry et al., 2024). These conversations included explicit references to the race of protagonists, responsibilities, and the perspectives of victims of racism. In contrast, color-blind discussions had no measurable effect on bias reduction. Therefore, it may be important to select children’s books and guide discussions in a way that more explicitly addresses the exclusion of specific outgroups.

However, it is also possible that the comparatively weaker intervention effects observed in our study were due to its less directive overall message, especially when compared to other stereotype-reduction programs. Interventions that communicate strong normative messages about the wrongness of intergroup exclusion may increase children’s self-presentational concerns, thereby affecting how they report their explicit outgroup attitudes (Wüthrich et al., 2024). It is therefore possible that interventions like ours show weaker effects because children are less concerned with impression management and therefore less likely to respond in socially desirable and strategic ways. This interpretation aligns with developmental research showing that children in middle childhood increasingly regulate their expression of intergroup attitudes in response to social norms and evaluative contexts (Monteiro et al., 2009; Rutland et al., 2005).

In accordance with previous research, we found that intervention effects were moderated by various child characteristics. First, we found a positive intervention effect on intergroup sympathy only for children without a migration background, not for those with a migration background. This finding aligns with the meta-analysis by Beelmann and Heinemann (2014), which showed that children from majority groups tend to benefit more from stereotype-reduction programs. One possible explanation is that majority-group children often hold more exclusive attitudes at baseline, and therefore have greater potential for attitudinal change (Killen & Rutland, 2011). Moreover, interventions may implicitly address majority-group norms and responsibilities, making their message more directly relevant and persuasive for children without a migration background (Beelmann & Heinemann, 2014).

Moreover, we found that reading involvement was positively associated with outgroup peer preference in the intervention group but not in the control group. This suggests that children who struggle to engage with reading may benefit less from reading-based interventions aimed at improving intergroup attitudes. To address this, future interventions should include strategies to enhance reading motivation, such as creative activities (e.g., staged readings) that foster immersion and identification with story characters—particularly among children who find reading less enjoyable (Kumschick et al., 2014; Schrijvers et al., 2019).

Finally, an intervention effect on children’s perceptions of intergroup classroom norms was only found for fourth graders, not for fifth graders. Similarly, Killen et al. (2022) found stronger effects of a DIY program on third graders compared to fifth graders. Yeager et al. (2015) reported decreasing effects of bullying intervention programs over adolescence. Compared to children, adolescents respond more negatively to the normative approaches of some intervention programs because they challenge adolescents’ social status in peer groups (Yeager et al., 2018). Even though our program was highly autonomy-supportive, with children taking leadership in discussions, it is possible that the children’s books were less appealing to the fifth graders in our study. Children’s motivation and valuation of narrative fiction decrease during the upper elementary grades (Parsons et al., 2018), while concerns about peers’ negative evaluations about reading increase (Webber et al., 2024). It is therefore possible that the fifth graders in our study responded less positively to children’s books and consequently benefited less from the literary discussions compared to the fourth graders.

### Limitations

This study has several limitations. First, we did not include a follow-up assessment. Findings from the 4Rs literary program suggest that literary interventions may show delayed effects on children’s social cognition (Jones et al., 2011). Therefore, we cannot determine whether similar sleeper effects occurred in our study—or whether any observed effects declined over time. Second, the study did not employ an active control group. As a result, we cannot rule out the possibility that the intervention effects were partly due to increased attention given to teachers and students by the research team. Third, the selected books covered a wide range of intergroup themes (e.g., exclusion based on gender, fictional outgroups, bystander behavior, inclusive group norms). In addition, high-quality literary texts rarely convey explicit moral messages; rather, they tend to be thematically complex and open to multiple interpretations. Consequently, the literary discussions could take diverse directions—some of which may have deviated from intergroup topics. While we see strong educational value in child-centered discussions, we also recognize the risk that some of them may have lacked the focus needed to bring about deeper changes in children’s outgroup attitudes.

Future research should address these limitations by (a) including follow-up assessments to track the stability and timing of effects, (b) implementing active control conditions matched on time and instructional demands to isolate specific program effects (Noble et al., 2019), and (c) developing shorter, more targeted interventions that use one or a few carefully selected children’s books focusing on particular minoritized groups, in order to test whether more focused units can more reliably foster positive intergroup attitudes.

### Implications

Despite its limitations, this study makes several contributions to theory, research, and practice. Using a relatively large sample and a cluster-randomized controlled design, it is, to our knowledge, the first study to demonstrate that a literature-based program integrated into regular language arts instruction can positively influence intergroup attitudes and inclusive classroom norms. The findings support theoretical accounts that emphasize the role of vicarious contact, perspective-taking, moral reasoning, and inclusive norms in shaping children’s evaluations of peers with a migration background, while also highlighting that such effects are small and not uniform across students.

For research, our results highlight the value of an interdisciplinary approach to designing and evaluating story-based intergroup interventions. Developmental and social psychology provide models of mechanisms underlying prejudice reduction, as well as conceptually and methodologically robust instruments to assess intergroup outcomes in children. Educational psychology, in turn, offers evidence on how these processes can be effectively supported in authentic classroom settings so that interventions foster both social and academic development. To more comprehensively capture the benefits of such integrated programs, future studies should jointly examine intergroup outcomes (e.g., attitudes, norms, peer relations) and academic outcomes (e.g., reading comprehension).

For educational practice, the study illustrates that promoting intergroup inclusion does not have to occur outside the curriculum. Embedding discussions of high-quality literary fiction into regular language arts instruction, using texts that resonate with curricular goals, may increase the feasibility and acceptability of such programs for schools and teachers. Using literary fiction rather than solely researcher-developed stories, and aligning social inclusion aims with existing learning goals such as reading comprehension, may facilitate more sustainable implementation of interventions for stereotype reduction in everyday classrooms. At the same time, the modest and selective effects observed here indicate that further refinement is needed to develop literary programs that reliably produce meaningful changes in intergroup inclusion.

## Supplementary Information

Below is the link to the electronic supplementary material.

Supplementary material 1 (DOCX 36.4 kb)

## Data Availability

The intervention materials were originally developed in German; illustrative sample materials are available at https://www.phbern.ch/skill. The analyses were not preregistered.
