# Supplementary material for: Promoting intergroup inclusion through literary discussions in fourth and fifth grades
Source: Soc Psychol Educ. 2026 Feb 27;29(1):21. doi: 10.1007/s11218-026-10179-0 (PMC13652880; doi:10.1007/s11218-026-10179-0)
Supplement: Supplementary file 1 [file 11218_2026_10179_MOESM1_ESM.docx]

1 **Intervention Modules**

The intervention consists of five modules: (1A) professional training on productive discussions, (1B) professional training on the books’ social themes, (1C) Quality Talk mini-lessons, (1D) book lessons, and (1E) teacher coaching.

**1A	Professional Training on Productive Discussions (September 2022)**

- **Dosage**: 5 hours
- **Facilitated by**: Third author
- **Objective**: This initial half-day training session focused on facilitating text-based discussions, drawing on principles from the *Quality Talk (QT) framework* (Murphy, 2018; Murphy et al., 2022).
- **Focus Areas**:
- Teachers reflected on their typically dominant role in classroom discussions and explored ways to foster more student-centered dialogue.
- Through video-based reflections of literary discussions, teacher enhanced their observation and reflection skills.
- Core elements of the QT framework were introduced, covering key aspects of classroom dialogues:

**(a) Quality of Questions**

- *Test Questions vs. Authentic Questions*: Test questions are closed-ended and factual and only require low-level thinking because they can be answered by referencing facts from the text. Authentic questions are open-ended and encourage higher-level thinking such as analysis, speculation, or generalization.
- *Types of Authentic Questions*: Adapted to literary and socio-cognitive contexts, five types were introduced to elicit socially and morally rich discussions: (1) *Big Questions* ask about moral dilemmas, main messages, or judgments about characters' decisions; (2) *Character Questions* explore mental states and social relationships of story characters; (3) *Affective Questions* invite emotional or personal connections to the story; (4) *Connection Questions* encourage intertextual links across different narratives; (5) *Uptake Questions* invite to build on peers’ ideas and promote elaboration. The first three question types replace the Speculation, Analysis, and Generalization questions from the QT framework. They were introduced to enhance opportunities for social and moral learning in discussions of literary fiction.

**(b) Response Quality**

- *Elaborative Answers*: Responses that support claims using multiple reasoning elements (e.g., reasons, textual evidence).
- *Cumulative Talk*: Contributions that build on each other without critical engagement.
- *Explorative Talk*: Exchanges where children challenge or critically evaluate differing perspectives.

**(c) Talk Moves**

- *Summarizing*: Recapping what has been said to maintain coherence in the discussion.
- *Modeling:* Demonstrating a discourse element explicitly (e.g., “I will ask an affective question…”).
- *Marking*: Praising the use of a productive discourse element (e.g., “Nice job asking a big question!”).
- *Prompting*: Encouraging reasoning and textual justification.
- *Challenging*: Inviting consideration of alternative perspectives.
- *Expanding*: Encouraging children to elaborate on peers' ideas.

**1B	Professional Training** **on the Books’ Social Themes (September 2022)**

- **Dosage**: 5 hours
- **Facilitated by**: First author
- **Objective**: The second half-day training focused on the social and moral themes embedded in the children’s books and introduced teachers to *Social Domain Theory* (Killen & Rutland, 2011; Smetana et al., 2014).
- **Focus Areas**:
- Teachers discussed how the selected books addressed different types of concerns—moral, group-based, and personal—and how these concerns conflict.
- They explored how children at this developmental stage typically reason about and respond to such conflicts.
- The training emphasized how to guide discussions that support children in coordinating and balancing conflicting concerns.
- Teachers were encouraged to help students make nuanced moral judgments while also considering contextual factors and mitigating circumstances of character’s immoral behaviors.
- Teachers engaged in video-based reflection on group dynamics during small-group discussions.
- They discussed strategies for creating an inclusive classroom climate and promoting equal participation, including techniques for handling with both dominant and shy students.

**1C	Quality Talk Mini Lessons (October–November 2022)**

- **Dosage**: 8 hours total
- **Led by**: Teachers & children
- **Objective**: The intervention began with a two-week series of nine mini lessons designed to teach children how to ask high-quality questions and respond effectively.
- **Lesson Structure**:
  1. **Explicit Introduction**: A PowerPoint slide introduced and defined the target discourse element.
  2. **Modeling**: A short, animated video clip demonstrated how to use the element in peer discussions.
  3. **Practice**: Students practiced the discourse element (e.g., brainstorming Big Questions after watching a short film).
- **Materials**: Teachers received detailed lesson plans and all teaching materials (e.g., PowerPoints, posters of QT questions and discussion rules).

**1D	Book Lessons (November 2022 – May 2023)**

- **Dosage**: 20 hours total (across all books)
- **Led by**: Teachers & children
- **Objective**: Over the school year, students read and discussed four children’s books in small groups. Discussions centered on peer inclusion/exclusion and were facilitated using the previously introduced discourse practices and Talk Moves.
- **Book Selection**:
- Chosen in collaboration with the Swiss National Children’s Literature Institute (see below for summaries and social themes of the selected books).
- All books or authors were nominees for important youth literature awards (e.g., the German Youth Literature Award).
- **Instructional Format**:
- Prior to reading, students discussed key themes (e.g., “What is bullying?”, “What are stereotypes?”) to build vocabulary and conceptual understanding.
- Books were read in two to three segments; audio versions were provided when needed.
- Students used *literacy journals* to note their authentic questions about the text, to encourage affective responses to the text (e.g., drawing scenes, describing characters’ mental states) and for work on their text comprehension (e.g., quiz on the story plot, tasks on vocabulary). Children also identified their individual goals for upcoming discussions (e.g., asking a question, provide one challenge).
- Small-group discussions lasted for 15 minutes and were guided by teachers using Talk Moves.
- Students reflected on their discussion goals after each session.

**1E	Teacher Coaching (November 2022 – May 2023)**

- **Dosage**: One coaching session per book
- **Facilitated by**: Four coaches, teachers, and a mastercoach
- **Objective**: Teachers received structured coaching based on the *MyTeachingPartner* format (Gregory et al., 2017) to support implementation fidelity and reflective practice. The four coaches had experience in facilitating discussions according to the QT approach. They participated in a one-day workshop and engaged in several peer consultation sessions during the intervention. The workshop and sessions were led by a mastercoach with extended coaching experience.
- **Coaching Process**:

1 Teachers recorded videos of their own literary discussions and submitted them to their coach.
2 Coaches selected short clips exemplifying effective questioning, responding, or facilitation of social group dynamics (e.g. encouraging peers to build on the contribution of a shy student).
3 Teachers completed written reflections using a structured *observation guide* that included indicators on discourse quality and positive social group dynamics.
4 Each coaching cycle included an online meeting where teachers and coaches discussed the selected clips and teachers’ written responses and planned the upcoming discussions.
2 **Thematic focus and summaries of the four children’s books**

**2A	Brown / Super-Bruno** (Øvreås & Torseter)

*Thematic focus*: Power imbalances, retaliation, group, inclusive friendships, and the role of adults in peer hierarchies.

*Summary:* Bruno’s grandfather has died, and shortly afterward, three older boys from the neighborhood destroy his hut. They keep hanging around in front of Bruno’s house, waiting for a chance to bully him. Bruno’s friends, Matze and Laura, stand by his side. Together with Bruno, Matze defends himself against the gang, and Laura offers them refuge. They are the only ones with whom Bruno talks about his grandfather’s death. At night, Bruno becomes a vigilante — as “Brauno,” he sneaks out and paints one of the older boys’ bicycles completely brown. During these nightly outings, he repeatedly meets his grandfather in his imagination, which helps him process his grief. When Matze finds out about Super-Bruno, he wants to join in and becomes “Schwarzke.” He paints the second bike black. Laura, now “Blaura,” also wants to help. The bullies and their parents begin to suspect Bruno, but they lack evidence. His father defends him, even though he has doubts. Fortunately, the three older boys are caught by the police vandalizing a church and are punished accordingly. With his problems now resolved, Bruno is able to say goodbye to his grandfather in a dream. He also puts away his superhero costume — he no longer needs it, as he has found true friends.

**2B	Rabbit & Bear: Attack of the Snack / Rotzhase und Schnarchnase** (Gough & Field)

*Thematic focus*: Origins of prejudice, intergroup threat, group conformity, and the social risks of challenging stereotypes.

*Summary:* A strange creature lands in the lake in front of Rabbit and Bear. As they observe it, they begin speculating about the potential danger of the little owl. The owl is unconscious and unable to defend herself. Rabbit spirals into a bizarre fantasy about the creature’s supposed wickedness. Although the woodpecker insists otherwise, Rabbit sticks to his accusations and drags other animals — a mouse and a mole — into his tirade of hate. They decide to build a prison for the owl. Bear tries to remind Rabbit of what actually happened and suggests waiting until the owl wakes up so they can get to know her. But when Rabbit refuses to listen, Bear has had enough and walks away. When the owl eventually wakes up in the prison, it becomes immediately clear to everyone that she is not a monster. Rabbit struggles to admit his mistake at first but ultimately feels relieved when he apologizes to the owl and the others. The other animals also recognize the injustice they committed. In the end, the animals come to understand that everyone has strange habits that may seem odd to others — and that this uniqueness makes each individual interesting and fascinating. The owl finally shows that she can make others laugh with her jokes. The story ends with the animals joyfully coming together in the forest, with humor emerging as the unifying element among the diverse woodland creatures.

**2C	Balaclava Boy / Tommy Mütze** (Robson)

*Thematic Focus:* Group conformity vs. individual privacy, bullying, ethnic/national diversity, gender stereotypes, and adult responses to exclusion.

*Summary*: Doogal and Dumisani live in South Africa and are best friends. At the beginning of the story, they are sitting in class 4SV, feeling bored by the lesson—until a new student, Tommy, is introduced. To everyone’s surprise, Tommy’s face is covered by a cap. Curiosity grows throughout the class, especially for Doogal and Dumisani. They become increasingly eager to find out why Tommy hides their face. They watch Tommy at lunch, repeatedly question them directly, offer their own theories, and even try to peek into Tommy’s home through the windows. Yet Tommy refuses to give any explanation, and the mystery remains. Despite this, both the friends and the entire class accept Tommy as part of the group. They particularly enjoy playing soccer with Tommy, who turns out to be an excellent player. Later, when older kids try to beat up Tommy during recess, some students from class 4SV come to the rescue. The class then decides to respect Tommy’s wish for privacy. The next day, they all show up at school wearing caps in solidarity. Feeling the support of the class, Tommy finally agrees to reveal the reason behind the cap. Tommy explains that they had to move frequently and used the cap to avoid being stared at as the “new kid.” Before taking off the cap in front of the whole class, Tommy asks for one thing: to continue playing soccer with the group. When Tommy removes the cap, the true reason is revealed — Tommy is a girl! The class cheers and celebrates her as their new soccer queen.

**2D	The Very Persistent Gappers of Frip / Die furchtbar hartnäckigen Gapper von Frip** (Saunders & Smith)

*Thematic focus:* Competition, meritocracy, social exclusion, moral disengagement, gender stereotypes, and resistance through fairness and cooperation.

*Summary:* Serena lives with her father and their goats in Frip, a small seaside town made up of just three houses. Since the death of her mother, Serena has taken care of not only the goats but also her father, who has resisted any further change in his life ever since. Their neighbors are Bea Romo and her two sons, Robert and Gilbert, and the Ronsen family with their daughters, Gloria and Beverly. For many years, the residents of Frip have struggled with a peculiar problem: Gappers. Gappers are tennis-ball-sized, orange creatures that live in the sea but move about on land. They are obsessed with goats and throw themselves onto them with a shrill cry of joy the moment they spot one. Once infested by Gappers, the goats stop producing milk, making them a serious nuisance. Every day, the children of the three houses must work hard to remove the Gappers from the goats and throw them back into the sea. The parents do not help, and neither do the neighbors support one another. One day, the smartest of the Gappers realizes that they could save time and effort if they focused only on the goats living closest to the sea. This insight leads them all to descend on Serena’s goats the next day, as her pasture is closest to the shore. For Serena, this means even more work and even fewer breaks. Her neighbors feel no obligation to help her. Instead, they interpret their own freedom from the Gapper problem as a sign of fate — believing that Serena and her father must somehow deserve this misfortune. They even write her a letter, stating that she is solely responsible for her situation and must resolve it herself. Lacking support from her father and her neighbors, Serena decides to sell the goats and take up fishing instead. When the Gappers find no goats in front of Serena’s house, they move on to the next closest house. This sparks a competition among the neighbors to see who can move their house furthest away from the sea — and who can afford to pay the most to do so. Soon, however, the neighbors run out of money and their relocated houses begin to sink into the swamp, unless they receive help. Serena notices their plight and decides to help them.

**References**

Gregory, A., Ruzek, E., Hafen, C. A., Mikami, A. Y., Allen, J. P., & Pianta, R. C. (2017). My Teaching Partner-Secondary: A video-based coaching model. *Theory Into Practice*, *56*(1), 38–45. https://doi.org/10.1080/00405841.2016.1260402

Killen, M., & Rutland, A. (2011). *Children and social exclusion: Morality, prejudice, and group identity*. Wiley-Blackwell. https://doi.org/10.1002/9781444396317

Murphy, P. K. (Eds.). (2018). *Classroom discussions in education*. Routledge.

Murphy, P. K., Croninger, R. M. V., Baszczewski, S. E., & Tondreau, C. L. (2022). Enacting Quality Talk discussions about text: From knowing the model to navigating the dynamics of dialogic classroom culture. *The Reading Teacher*, *75*(6), 717–731. https://doi.org/10.1002/trtr.2110

Smetana, J. G., Jambon, M., & Ball, C. (2014). The social domain approach to children’s moral and social judgments. In *Handbook of Moral Development*. Routledge. https://doi.org/10.4324/9780203581957.ch2
